# Hemolysis From Intravenous Immunoglobulin in Obese Patients With Kawasaki Disease

**DOI:** 10.3389/fped.2020.00146

**Published:** 2020-04-03

**Authors:** Khanh-Van Y. Van Anh, Saloni Shah, Adriana H. Tremoulet

**Affiliations:** ^1^School of Medicine, Tulane University, New Orleans, LA, United States; ^2^Department of Pediatrics and Rady Children's Hospital San Diego, University of California, San Diego, La Jolla, CA, United States

**Keywords:** Kawasaki disease (KD), intravenous immunoglobulin (IVIG), hemolytic anemia, obesity, lean body mass

## Abstract

**Objective:** We assessed the risk of IVIG-associated hemolytic anemia in patients with acute Kawasaki disease (KD) and evaluated the risk of weight-based dosing in our obese patients.

**Methods:** IVIG-associated hemolytic anemia was assessed in acute KD patients treated with IVIG at Rady Children's Hospital-San Diego. Patients in whom hemolytic anemia was suspected had a decrease in z-score of their hemoglobin (zHgb) at least two standard deviations below the cohort's mean change in zHgb from baseline to 2 weeks post-IVIG treatment. These patients were further evaluated for spherocytosis, blood type, need for transfusion, red cell distribution width, reticulocytosis, and direct Coombs test. Body mass index was calculated.

**Results:** Of the 30 IVIG-resistant KD patients who received a second dose of IVIG, 2 (6.7%) developed hemolytic anemia after a total of 4 g/kg of IVIG dosed on actual body weight, or a mean of 4.6 g/kg of IVIG based on lean body mass. Compared to 496 non-obese KD patients who received a single dose of IVIG with no cases of hemolytic anemia, two (5.6%) of 36 obese KD patients developed hemolytic anemia after a single dose of IVIG (2 g/kg) dosed on actual body weight, or a mean of 2.7 g/kg IVIG based on lean body mass.

**Conclusions:** In addition to following patients carefully for hemolytic anemia after a second dose of IVIG, physicians should consider IVIG dosing based on lean body mass for obese patients.

## Introduction

Kawasaki disease (KD) is an acute vasculitis in children that if left untreated can lead to coronary artery aneurysms in 25% of patients (1). If treated with intravenous immunoglobulin (IVIG), the rate of coronary artery aneurysms decreases to 5% (2). The recommended treatment for patients with acute KD is a single high-dose (2 g/kg) IVIG infusion within 10 days of illness onset (3). Common adverse effects of IVIG in acute KD patients include mild effects such as headache and mild anemia acutely following treatment, as well as more serious effects such as thrombosis, anaphylaxis or hemolysis requiring transfusion (4, 5). The complication of antibody-mediated hemolytic anemia is increased in patients receiving a high cumulative dose of IVIG, and thus increases in patients given a second dose of IVIG for treatment-resistant KD (6–9). The risk is also higher in patients with non-O blood group. Cases of IVIG-related hemolytic anemia have been reported in the treatment of various other conditions including immune-mediated thrombocytopenia, Guillain-Barre syndrome, and myasthenia gravis (10). The risk of hemolysis appears to be higher in pediatric patients treated with two doses of 2 g/kg of IVIG for KD than in pediatric and adult patients treated for chronic inflammatory or immunodeficiency disorders (9). Rates of hemolytic anemia in patients with KD related to IVIG treatment have varied from 0.06 to 16% after a single dose of IVIG, and as high as 44% in KD patients treated with a second IVIG infusion (5, 7, 9–11). The concern for hemolytic anemia was confirmed in a post-marketing surveillance study in which 39% of IVIG-associated hemolytic events in pediatric patients were in children with KD (10). Given this concern, we assessed the occurrence of suspected IVIG-associated hemolytic anemia in KD patients treated at our center, with a focus on comparing rates of hemolytic anemia in obese and non-obese KD patients.

## Materials and Methods

IVIG-associated hemolytic anemia was assessed utilizing the Rady Children's Hospital-San Diego (RCHSD) Pharmacy Clarity reporting system, Research Electronic Data Capture (REDCap) KD database, and medical records of the acute KD patients treated at RCHSD between January 1, 2010 and January 31, 2018. The following clinical data were prospectively collected for the 661 acute KD patients treated with IVIG at RCHSD: patient demographics, illness day at diagnosis (illness day 1 = first day of fever), response to IVIG therapy, and coronary artery status (Z scores, echocardiographic measurements of the internal diameter normalized for body surface area of the proximal right coronary artery and left anterior descending coronary artery). Weight, height and age were used to calculate body mass index (BMI), and a BMI of >95% for age was considered obese (12). We retrospectively recorded details of the IVIG infusion including lot number and concentration of IVIG administered and time and duration of IVIG infusion. All patients received Gammagard (Baxter) product as either the 5 or 10% solution. Fever was defined as rectal or oral temperature ≥38.0°C; patients with persistent or recrudescent fever ≥36 h after completion of IVIG were classified as IVIG-resistant (3). Patients in whom hemolytic anemia was suspected had a decrease in zHgb ([Observed hemoglobin-Mean hemoglobin for age]/Standard deviation for age) of at least two standard deviations below the cohort's mean change in zHgb from the acute to the subacute phase (median = 14 days post-IVIG treatment, IQR = 12–16.5). These patients were further evaluated for spherocytosis, blood type, need for a blood transfusion, red cell distribution width, reticulocytosis, and direct Coombs test when available. Due to the retrospective nature of this study, the direct Coombs test, serum haptoglobin, reticulocyte count, and indirect bilirubin levels were unavailable for definitive identification of hemolytic anemia in many patients. Fisher's exact test was used to compare the rates of IVIG-associated hemolytic anemia in obese and non-obese patients.

The study protocol was reviewed and approved by the University of California San Diego's Institutional Review Board. Written informed consent was obtained from a parent or legal guardian, and assent, when appropriate, was obtained from the patient.

## Results

**Figure 1:**
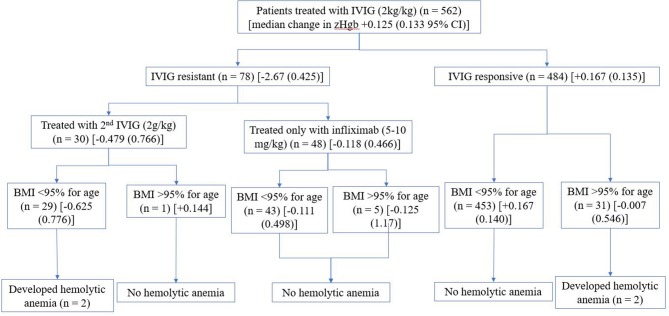
Summary of cohort [median change in zHgb (95% confidence interval)].

Of the 661 acute KD patients treated with IVIG (2 g/kg), 99 received infliximab initially as either part of a clinical trial or because of significant coronary artery damage or KD shock (13). Of the remaining 562 IVIG-treated KD patients who were initially treated with single dose of IVIG, 78 (13.9%) were IVIG-resistant. Of these IVIG-resistant KD patients, 9 (11.5%) received a second IVIG infusion (2 g/kg), 48 (61.5%) received infliximab (5–10 mg/kg), and 21 (26.9%) received both a second dose of IVIG (2 g/kg) and infliximab (Figure 1). Hemolytic anemia was defined as a drop in zHgb of at least −3.17 (2 SD below the mean drop in zHgb of −0.024 in this study population) between the acute and subacute phase and the presence of at least polychromasia and anisocytosis on the peripheral smear. Of the 30 IVIG-resistant acute KD patients who received a second IVIG infusion, two (6.7%) developed hemolytic anemia.

The first patient developed hemolytic anemia after receiving 76.4 g (4 g/kg) of IVIG. This patient was admitted with a baseline hemoglobin level of 11.4 g/dl (zHgb of −1.125), which dropped to 10.7 g/dl (zHgb of −2) following the first IVIG infusion, and 7.7 g/dl (zHgb of −5.75, change in zHgb of −4.625 from the pre-IVIG value) after the second IVIG infusion. This patient had a BMI of 16.2 kg/m^2^ (68.8% for age). Spherocytosis and anisocytosis were present on the blood smear. Blood type was not determined, and direct Coombs test was not performed as the patient did not require a blood transfusion and the hemoglobin increased to 11.8 g/dl within a month after treatment.

The second patient developed hemolytic anemia after receiving 90.4 g (4 g/kg) of IVIG. This patient was admitted with a baseline hemoglobin level of 11.3 g/dl (zHgb of −2), which dropped to 10.6 g/dl (zHgb of −2.17) following the first IVIG infusion, and 6.1 g/dl (zHgb of −7.78, change in zHgb of −5.78 from the pre-IVIG value) after the second IVIG infusion. This patient had a BMI of 13.4 kg/m^2^ (4.7% for age). Spherocytosis, anisocytosis, and polychromasia were noted on the blood smear consistent with hemolytic anemia. The reticulocyte count was elevated to 7.1%. This patient was blood type A and was transfused 15 cc/kg of O- blood with prompt improvement in clinical symptoms of fatigue and dizziness as well as an increase in hemoglobin to 11.7 g/dl. Direct Coombs test was not performed and therefore unavailable for analysis. Given recrudescence of fever, this patient then received a dose of infliximab (10 mg/kg) without worsening of the anemia. Of the 48 KD patients who received only infliximab for IVIG-resistance, none developed hemolytic anemia.

As compared to KD patients who received two doses of IVIG, only two of the 532 (0.4%) KD patients who received a single dose of IVIG developed hemolytic anemia and both met the definition for obesity with a BMI at or above the 95th percentile for age (12). Of the 532 KD patients who received a single dose of IVIG, 36 (6.6%) were obese. Thus, the rate of hemolytic anemia in KD patients receiving a single dose of IVIG was significantly higher in our obese KD patients (2/36, 5.6%) as compared to non-obese KD patients (0/496, 0%; *p* = 0.0045, although confounded by small numbers). These 36 obese patients had a median change in zHgb of −0.125 (0.49 95% CI) between the acute and subacute phase as compared to a median change in zHgb of +0.167 (0.135 95% CI) in the 496 non-obese patients (*p* = 0.23).

The first obese patient developed hemolytic anemia after receiving 68 g (2 g/kg) of IVIG. This patient was admitted with a baseline hemoglobin of 12.5 (zHgb of −0.22), which dropped to 9.1 (zHgb of −4, change in zHgb of −3.78) 2 days after a single dose of IVIG (2 g/kg). At the age of 6 years and 10 months, this patient met the definition of obese with a weight of 34 kg (98.5% for age), a height of 127 cm (86.9% for age), and thus a BMI of 21.1 kg/m^2^ (98.3% for age). Moderate anisocytosis was seen on the peripheral blood smear. The red blood cell distribution width was 17.5%, consistent with hemolytic anemia. Blood type was not determined, and direct Coombs test was not performed as the patient was not symptomatic. The hemoglobin increased to 13.9 (zHgb of 1.33) 3 weeks after receiving IVIG.

The second obese patient developed hemolytic anemia after receiving 134 g (2 g/kg) of IVIG. This patient was admitted with a baseline hemoglobin of 13.6 (zHgb of 0.56), which dropped to 9.3 (zHgb of −4.22, change in zHgb of −4.78) 11 days after a single dose of IVIG (2 g/kg). This was associated with clinical symptoms of light-headedness and fatigue. At the age of 11 years, this patient met the definition of obese with a weight of 67 kg (99.2% for age), a height of 149.1 cm (77.4% for age), and thus a BMI of 31 kg/m^2^ (99.1% for age) (12). Moderate polychromasia and anisocytosis were seen on the peripheral blood smear. The reticulocyte count and red blood cell distribution width were elevated at 11.5 and 22%, respectively, consistent with hemolytic anemia. This patients' blood type was A+. Direct Coombs test was not performed. Screening of the lot of the IVIG product administered to this patient was conducted by the RCHSD Blood Bank for titers of anti-A and -B antibodies. A manual tube method with 3-5% of reagent red blood cells (Quotient Biodiagnostics) showed the following: A1 cells 3+, A2 cells 2+, and B Cells 1+, suggesting the presence of high titer of anti-A antibodies. Subgroups of A blood types, including blood type A2, have been associated with anti-A1 antibodies which can react with A1 cells (14). The hemoglobin normalized without transfusion and was 12.7 g/dl 6 weeks after treatment for KD.

## Discussion

We report four cases of hemolytic anemia following IVIG therapy for acute KD. Two of these patients had hemolytic anemia after two doses of IVIG (6.7% rate of hemolytic anemia) while two obese patients developed hemolytic anemia after a single dose of IVIG (5.6% rate of hemolytic anemia in obese patients). None of the 48 patients treated with infliximab for IVIG-resistant KD developed hemolytic anemia. The addition of infliximab prior to treatment with IVIG has been shown to shorten fever duration, more rapidly reduce inflammation and eliminate the risk of transfusion reactions if given before IVIG, but has shown no significant difference in adverse events, such as hemolytic anemia, following IVIG (13).

IVIG causes hemolysis either via isohemagglutinins in the IVIG product administered, or by enhanced erythrocyte sequestration via activation of the complement pathway by the IgG complexes, leading to erythrophagocytosis and a reduction in hemoglobin (10, 15). Hemolysis has only been reported in patients with blood group O in exceptional cases while the frequency of hemolytic events in patients with blood group AB exceeded the frequency of patients with blood group A or B (10). Patients with blood group AB had a ratio of cases to population distribution of 2.6–9 compared to 1.8–2.8 for blood group A, 0.3–0.7 for blood group B, and 0.05–0.08 for blood group O (10). Of the four patients with hemolytic anemia in our study, two were blood type A while the other two were unknown. As with previous reported cases of hemolytic anemia, our patients had spherocytosis, polychromasia and an elevated reticulocyte count, and the two patients who received a second dose of IVIG had an abrupt drop in hemoglobin concentration following the second infusion (6–9). One patient had an A+ blood type and a high level of anti-A antibody in the lot of IVIG with which he was treated. Isoagglutinin reduction measures such as anti-A donor screening and anti-A/anti-B specific immunoaffinity chromatography in IVIG manufacturing have been predicted to reduce the risk of hemolysis in patients with non-O blood groups, especially those with blood group AB (16).

While the rate of hemolytic anemia after IVIG correlates with increasing doses of IVIG, little is known about the risk of hemolytic anemia in obese patients as dosing is based on absolute weight. A study of cases of IVIG-associated hemolysis over 10 years found that IVIG-associated hemolysis tends to occur in patients who received doses exceeding 2 g/kg body weight (60% of cases), as well as in patients with comorbidities such as hypertension or anemia (10). Childhood obesity is associated with comorbid metabolic risk factors such as hypertension, type 2 diabetes, and abnormal lipid profiles (17). Obese children are at a three-fold higher risk for hypertension than non-obese children (18). The two obese patients who developed hemolytic anemia received 68 g of IVIG based on a weight of 34 kg and 134 g of IVIG based on a weight of 67 kg. Had the 2 g/kg dose of IVIG been based on estimated lean body mass of 26.6 and 46.4 kg, they would have received 53.2 and 92.8 g of IVIG. Instead, based on lean body mass, these two obese patients received 2.6 and 2.9 g/kg of IVIG, respectively (19).

The two non-obese patients who developed hemolytic anemia after a second dose of IVIG each received an equivalent of 4.7 and 4.4 g/kg of IVIG respectively, based on lean body mass. The 496 non-obese patients who received a single dose of 2 g/kg of IVIG received on average 2.3 g/kg (*SD* = 0.12) of IVIG based on lean body mass. The 36 obese patients who received a single dose of IVIG received on average 2.6 g/kg (*SD* = 0.15) of IVIG based on lean body mass. As IVIG is distributed primarily in the vascular space because it is a relatively polar molecule, plasma concentrations of IVIG, and consequently anti-A antibodies, are higher for obese as compared to lean patients if IVIG is dosed on actual body weight (20, 21). There have been several other case reports of thrombotic and hemolytic complications following IVIG treatment in obese patients with primary immunodeficiency disorders, possibly due to large doses of IVIG (22, 23). Other studies have also shown that the correlation of serum immunoglobulin levels is strongest with doses based on ideal or adjusted body weight rather than actual body weight in obese patients (21, 24). While several studies have discussed the cost-saving benefits of weight-adjusted dosing of IVIG in obese patients (25, 26), only a limited number have reported on tolerability or safety related outcomes. One retrospective multicenter study of 297 adult patients treated with IVIG for any indication, the most common being neuroimmunological disorders, showed no increase in 30-day hospital readmission or length of stay with ideal body weight-based dosing of IVIG compared with total body weight dosing (27). Another study also showed that an individualized IVIG treatment protocol using a dosing weight calculated from the patient's ideal body weight is clinically non-inferior to standard dosing for chronic inflammatory demyelinating polyneuropathy, and is 10–25% more cost-effective (28). Thus, this raises the issue as to whether obese KD patients should be dosed based on estimated lean body mass rather than actual body weight.

This study had several strengths and weaknesses. This study included a large cohort of acute KD patients evaluated for hemolytic anemia post-IVIG. In addition, the use of either infliximab or second IVIG for IVIG-resistant KD allowed comparison of rates of hemolytic anemia following these two treatments. This is also the first study to compare the rates of hemolytic anemia in obese and non-obese populations following IVIG treatment for Kawasaki disease. Limitations included small number of cases of hemolytic anemia limiting the ability to perform statistical analyses, missing laboratory data for assessment of hemolytic anemia, and the ability to only assess one of the four IVIG aliquots administered for anti-A/anti-B antibodies. Thus, we may have underestimated the rate of IVIG-associated hemolytic anemia. The KIDCARE trial, a nationwide study comparing a second dose of IVIG vs. infliximab for IVIG-resistant KD will provide prospectively collected, standardized data on the prevalence of hemolytic anemia following second IVIG infusion (NCT #03065244) (29). Physicians should be aware of hemolytic anemia as a potential treatment complication, particularly in KD patients treated with a second infusion of IVIG and need to consider lean body mass dosing for obese patients.

## Data Availability Statement

The datasets generated for this study are available on request to the corresponding author.

## Ethics Statement

This study protocol was reviewed and approved by the University of California San Diego's Institutional Review Board. Written informed consent was obtained from a parent or legal guardian, and assent, when appropriate, was obtained from the patient.

## Author's Note

The data were presented at the NIH Short-Term Research Training Grant annual conference at the University of California, San Diego, in La Jolla, California.

## Author Contributions

K-VV contributed to the conception and design of the work, data collection, data analysis and interpretation, and writing of the manuscript. SS contributed to the conception and design, data collection, data analysis, and interpretation, and drafting of the manuscript. AT was responsible for the conception and design of the project, supervision of the work, and provided critical revision of the manuscript. All authors contributed to the final manuscript.

### Conflict of Interest

The authors declare that the research was conducted in the absence of any commercial or financial relationships that could be construed as a potential conflict of interest.
